# Choice architecture interventions promoting sustained healthier food choice and consumption by students in a secondary school setting: a systematic review of intervention studies

**DOI:** 10.1017/S1368980023001118

**Published:** 2023-09

**Authors:** Eva Andrea Schulte, Gertrud Winkler, Christine Brombach, Anette E Buyken

**Affiliations:** 1 Public Health Nutrition, Institute of Nutrition, Consumption and Health, Paderborn University, Paderborn 33098, Germany; 2 Department of Life Sciences, University of Applied Sciences Albstadt-Sigmaringen, Sigmaringen, Germany; 3 Forschungsgruppe für Lebensmittel-Sensorik, Life Sciences and Facility Management, Züricher Hochschule für Angewandte Wissenschaften, Zürich, Schweiz

**Keywords:** Choice architecture, Nudge, High school setting, Adolescence, Effectiveness, Sustainability, Food choice

## Abstract

**Objective:**

To systematically review the impact of choice architecture interventions (CAI) on the food choice of healthy adolescents in a secondary school setting. Factors potentially contributing to the effectiveness of CAI types and numbers implemented and its long-term success were examined.

**Design:**

PUBMED and Web of Science were systematically searched in October 2021. Publications were included following predefined inclusion criteria and grouped according to the number and duration of implemented interventions. Intervention impact was determined by a systematic description of the reported quantitative changes in food choice and/or consumption. Intervention types were compared with regard to food selection and sustained effects either during or following the intervention.

**Setting:**

CAI on food choice of healthy adolescents in secondary schools.

**Participants:**

Not applicable.

**Results:**

Fourteen studies were included; four randomised controlled trials and five each of controlled or uncontrolled pre–post design, respectively. Four studies implemented a single CAI type, with ten implementing > 1. Three studies investigated CAI effects over the course of a school year either by continuous or repeated data collection, while ten studies’ schools were visited on selected days during an intervention. Twelve studies reported desired changes in overall food selection, yet effects were not always significant and appeared less conclusive for longer-term studies.

**Conclusions:**

This review found promising evidence that CAI can be effective in encouraging favourable food choices in healthy adolescents in a secondary school setting. However, further studies designed to evaluate complex interventions are needed.

Childhood and adolescence are considered formative years for food preferences that tend to persist into adulthood^(1,2)^. Upon entering adolescence, peers, current food trends and the surrounding food environment gain progressively more influence on adolescents’ food choices^(3)^. This commonly entails a shift towards less favourable eating habits associated with a lower fibre, fruit and vegetable intake, higher intake of energy-dense foods, fat and sugar and more frequent snacking. The last is presumably due to the omnipresence of convenience and highly processed food, often targeted to appeal specifically to this age group. Effective and sustainable strategies to counteract these unfavourable changes in eating habits among adolescents are thus considered vital for an individual’s healthy development and well-being in later life^(2)^. As adolescents are highly susceptible to environmental influences on food choices, choice architecture interventions (CAI) present a promising tool to promote healthier food choices and eating habits in this age group^(4–6)^. CAI are based on the concept that food choices are often the result of habits and patterns involving emotional (and minimal conscious) thought in decision-making^(7)^. By changing favourable food options’ position and/or properties, thus increasing their salience and/or convenience within a given food environment, CAI aims to encourage unconscious choice of healthier *v*. less favourable food options without actually limiting overall food range available or limiting free choice^(8,9)^. As adolescents spend a considerable part of their day in school, where they consume one or several of their meals, school cafeterias and lunch rooms appear as ideal settings for CAI implementation and promoting the establishment of healthier food choice habits^(10)^.

We aimed to carry out a systematic review of existing literature on interventions for healthy adolescents’ food choices using CAI in a secondary school setting to evaluate the impact of such changes in respective food environments in terms of effectiveness, while differentiating between CAI types as well as respective number of implemented interventions. Additionally, we attempt to determine sustained CAI effects as reported food selection changes could result primarily due to a form of novelty effect^(11)^.

Based on our findings, we would like to propose possible recommendations for the successful CAI implementation for our targeted age group and setting as well as suggest future areas for associated research. Thus, this review aims to answer the question whether CAI can be an effective strategy in promoting adolescents to change existing eating patterns and nutritional choices within a secondary school setting in a lasting way and which factors might contribute to its continuous success.

## Method

### Selection criteria

To evaluate sustained CAI effectiveness, only quantitative studies in a secondary school setting (i.e. middle, high, or intermediate school, depending on the country and school system) were included in this review. Studies including children from primary schools or students from universities, vocational schools and/or colleges were not included unless the data also included secondary school settings and was analysed in sub-groups, allowing extraction of the secondary school setting data. A similar approach was used for studies reporting on additional educational and/or exercise programmes in combination with CAI. Studies were excluded if only the interaction effect of CAI with the additional programmes was reported. Additionally, studies with a particular focus on a specific subset of secondary school students concerning gender, ethnicities, religion, learning abilities, health and/or social status or studies aimed at specific school environments were also excluded. On account of the selected setting, students’ age ranged from 10 to 12 up to 18 years, depending on the school system of the country in which the study was conducted, corresponding to the WHO definition of adolescence^(12)^ and encompassing the formative years for developing and consolidating major food preferences and eating habits^(1,2)^.

### Definition of terms

Favourable/healthy nutritional choices comprise all provisions of foods, beverages and meals higher in nutrient density, lower in energy, salt, sugar, cholesterol and fat as well as less processed items. Based on such choices, students should choose, purchase and consume more vegetables, fruits, less meat, fat, sweets and sugar-sweetened beverages, more whole-grain and unsweetened food and beverages and more water^(13)^.

According to the CAI definition by the TIPPME (typology of interventions in proximal physical micro-environments)^(8,9)^, all studies included in this review had to change position/placement and/or properties of objects and stimuli within a given food microenvironment without limiting or restricting the students’ choices. A detailed description of CAI parameters used for this review is presented in Table 1. Studies were excluded that directly influenced students’ choice, e.g. via free food programmes or other monetary benefits. This was also applied to studies that restricted the students’ choice, e.g., by removing unfavourable food and/or beverages from the school, as well as any study where the fundamental quality (such as energy density, fat content or palatability) of the food was changed as part of the intervention. Food environments in a school setting included the school cafeteria, canteen, lunchroom, as well as vending machines or concession stands at school events. The search term ‘food’ includes food and/or beverage.

**Table 1:** Overview of the classification of CAI implemented in the included studies based on the TIPPME typology^(8,9)^

Intervention class	Intervention type	Definition	Examples	Studies/examples
Primarily alter properties of product or related object	Functionality/ Functional design	physical changes of equipment or products that change how they work	Shape/type/size of eating/drinking utensils, e.g. easier to open, pour or access	29,30
	Information/Labelling	Changing words, symbols or pictures on product or related objects that convey information about the product or object	• Nutritional labels about nutrients, calories • Traffic lights, health warnings • Fruit/vegetables with nice and interesting names	19,21–26,28–32
	Presentation	Sensory quality and/or visual design of product or its packaging is altered	Way food is arranged, cut, shaped, e.g. see-through containers, pre-sliced food, fruit/vegetables in nice bowls/containers	20,23,25,26,28,30–32
	Sizing^*^	Size or quantity of product or related object is changed	• Package, portion size • Size of product, e.g. smaller plates	–
Primarily alter placement of product or related objects	Availability	Adding behavioural options to environment with previous potential behaviour still available	Increasing available healthy food option, e.g. more healthy items in vending machines, greater variety of fruit/vegetables in cafeteria	25,27–32
	Position/ Proximity	Accessibility, visibility, proximity of product is altered thus reducing effort for choosing healthier options by making them more salient	• Decreasing distance of products from routes of passage, e.g. fruit/vegetables next to cash register • Placing certain products less prominently • Changing item position on menu	23,25–28,30–32
Alter both properties and placement of product or related objects	Priming	Placement of stimuli within micro-environment to influence via the activation of a semantic relationship or associative process	• Placing decorative objects within a lunchroom or cafeteria	25,30
	Prompting	Explicit verbal, visual, numeric information to promote awareness, more general motivational prompting	• Motivational prompts, e.g. footsteps, posters signs • Promotional signs, e.g. ‘Eat more fruit!’	23–25,30

* None of the studies matching the inclusion criteria for this review implemented this intervention type.

Implemented CAI impact was evaluated by reported quantitative changes occurring in food choice and/or consumption by students during an intervention. The term ‘pre-post design’ was assigned to any study comparing baseline data prior to the implementation of CAI with that during the intervention itself. Sustainability of changes was assessed from studies that compared data from different time points during the intervention (and/or after changes were removed) with that prior to CAI implementation.

### Search strategy, data extraction and synthesis

**Fig. 1:**
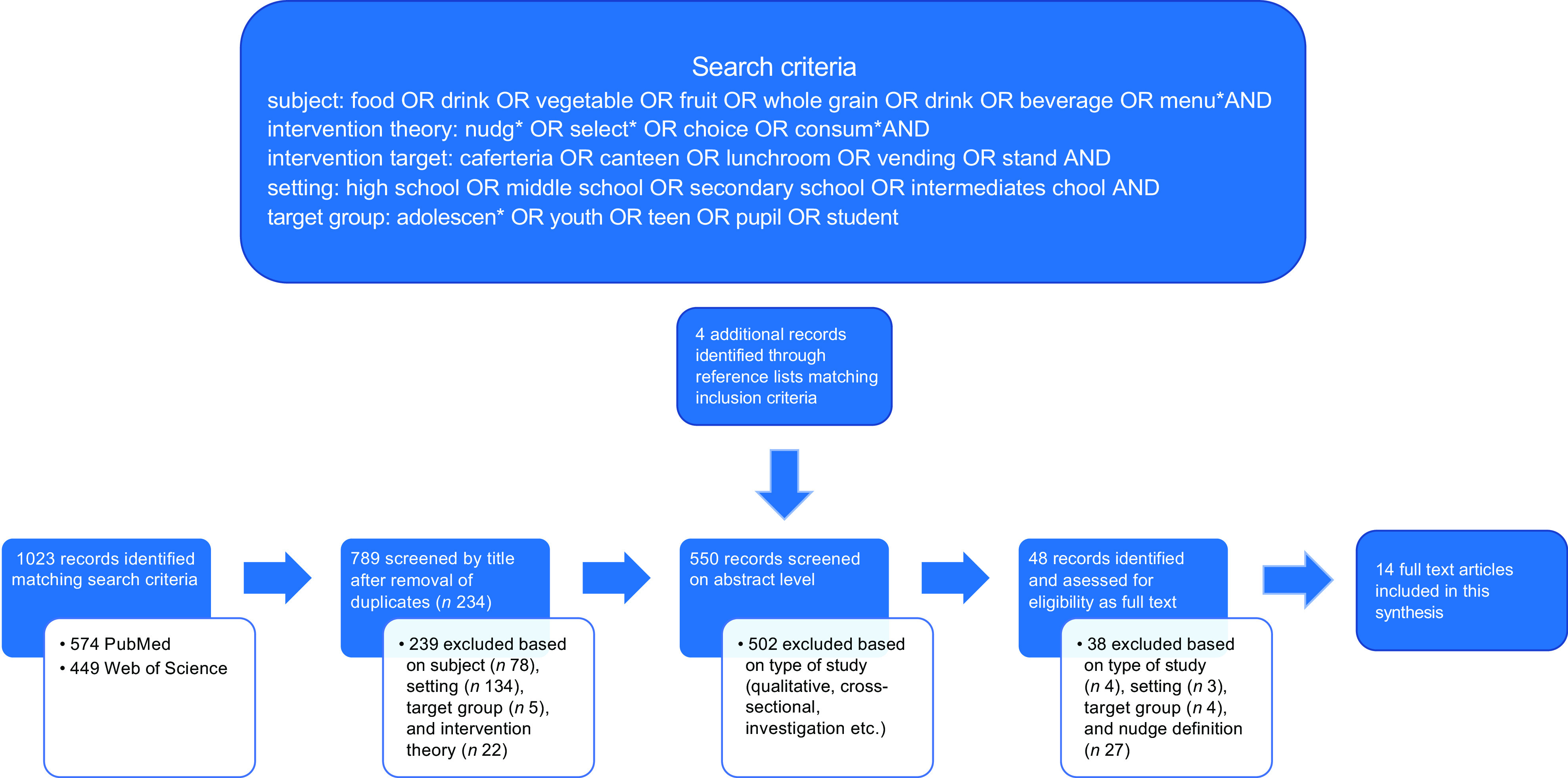
Overview of search criteria and strategy

Authors AEB and EAS systematically searched the electronic databases PUBMED and Web of Science in October 2021 using the search criteria shown in Fig. 1 for subject, intervention theory, intervention target, setting and target group, respectively. No limit was placed on the publication date, with languages limited to English and German. Following the retrieval of 789 records and removal of duplicates, publications were reviewed independently by AEB and EAS at title and abstract level. Four additional records were identified through the screening of reference lists of related articles and reviews by EAS^(14–18)^. A total of fifty-two records met the inclusion criteria, or did not meet the exclusion criteria, and were assessed for eligibility in full. Finally, based on the selection criteria stated above, AEB and EAS unanimously agreed on fourteen studies matching the selection criteria included for this qualitative synthesis. Data extraction was initially performed by EAS and verified for accuracy by AEB. Due to heterogeneity among study designs, intervention types and measured parameters, a meta-analysis could not be performed. A narrative synthesis was used to evaluate the studies included in this review, with CAI types, measured parameters, outcomes and main results summarised and tabulated to provide an overview. Studies were additionally divided by the number of interventions implemented (one or multiple) as well as the duration of intervention and time points of result reports during or after the intervention.

The quality of the study design was judged based on a procedure proposed by Sanson-Fisher et al., who rated the study design based on its ability to answer three methodological questions (1) has a change occurred (2) was the change due to the intervention (3) is the degree of change significant. Based on their assessment, a cluster randomised trial design is rated as having a strong ability for all three questions, a controlled pre–post design as having a moderate ability for all three questions and an uncontrolled pre–post design to have a moderate ability for questions 1 + 3 and a weak ability to answer question 2. Hence, we judged the overall quality of cluster randomised controlled trials as strong, controlled pre–post design as moderate and uncontrolled pre–post designs as weak-to-moderate (see Table 2).

**Table 2:** Overview of study design of included studies

Reference/ country	Study Design^†^/overall quality of design	Setting	Control	Duration
Conklin MT et al. 2005^(19)^ PA, USA	Controlled pre-post / moderate	6 public high schools, N = NR	✓ 2 control/ 4 intervention schools	total of 12 weeks for two meal cycles, 6 weeks baseline, 6 weeks intervention
Wansink B et al. 2013^(20)^ NY, USA^*^	Cluster RCT^‡^^/^strong	6 public middle schools *n* 2150 students	✓ 3 control/ 3 intervention schools	1-month intervention; selective data collection at 2 d during each period
Hunsberger M et.al. 2015^(21)^ OR, USA	Uncontrolled pre-post / moderate-to-weak	1 rural middle school; *n* 531 students		data collection at 17 school days each during baseline (Jan 2010) and intervention (Feb 2010)
dosSantos et al. 2018^(22)^ Denmark	Controlled pre–post / moderate	3 public schools *n* 97 students	✓ 2 control (food was catered in lab at university)/1 intervention schools	4 d from Feb to May 2017
Hanks AS et al. 2013^(23)^ NY, USA^*^	Uncontrolled pre–post/ moderate-to weak	2 public high schools; *n* 3762 students		2 months baseline 2 months intervention, data collection at 4 d during each period
Cullen KW et al. 2015^(24)^ TX, USA	Cluster RCT / strong	4 intermediate schools *n* 427 students	✓ *n* 2 control schools with 215 students, *n* 2 intervention schools with 215 students	1 d per week during fall semester 2011 observing 3–4 students per visit
Greene KN et al. 2017^(25)^ NY,USA	Cluster RCT/strong	10 middle schools; *n* 7752 students	✓ 3 control/ 7 intervention schools (4 fruit, 3 vegetable, one dropped out)	1 month baseline, 2 months intervention, selective data collection at 5 d during baseline and 4 d during the intervention
Quinn EL et al. 2018^(26)^ WS, USA	Controlled pre–post / moderate	11 sary schools; *n* 2309 students	✓ 5 control/ 6 intervention schools	1 visit at the beginning of the year, 1 visit at the end of the intervention year
Hanks AS et al. 2012^(27)^ NY, USA	Uncontrolled pre–post/ moderate-to-weak	1 public high school; pre *n* 602 students intervention *n* 482 students		8 weeks baseline, 8 weeks intervention; selective data collection at 2 d during each period
Boehm R et al. 2020^(28)^ Northeast, US	Controlled pre–post/ moderate	2 high schools *n* 3437 students	✓ 1 control/1 intervention school	baseline Sept–April, intervention 4 weeks after spring break 282 d baseline, 50 d intervention in total
Kenney, EL et al. 2015^(29)^ NY, US	Cluster RCT / strong	10 public schools; N = NR	✓ 5 control/5 intervention schools	1 school week at baseline (April 2013) and follow-up (May–June 2013) about 3 weeks afterwards
Winkler G et al. 2018^(30)^ Bavaria, Germany	Uncontrolled pre-post / moderate-to-weak	1 high school (Gymnasium); *n* 1300 students		1 year, data collection in four phases prior (1), right after implementation (2), after 5 months (3), after 1 year (4); 15–16 d of data collection during each phase
Koch PA et al. 2020^(31)^ NY, US	Uncontrolled pre–post / moderate-to-weak	3 public high schools; *n* 899 pre-redesign, *n* 1193 3-month post-redesign, *n* 1222 1-year post-redesign		2 consecutive days pre-, 3 months and 1-year post-redesign
Ensaff H et al. 2015^(32)^ Yorkshire, UK	Controlled pre–post / moderate	2 sary schools; *n* 980 students	✓ 1 control/ 1 intervention school	1 year, 190 school days per academic year, pre (29 weeks), intervention (6 weeks), post (3 weeks)

Light grey = studies implementing one intervention only.

Medium grey = studies reporting results for different time points during or after intervention.

* Studies from this group have provoked criticism as can be seen at https://peerj.com/preprints/3137.pdf. As a result, some studies had to be retracted. However, any study cited in this review has not been retracted at the point of writing, though results should be considered with care.

† Terminology based on Sanson-Fisher^(37)^; the term *pre–post* design is used for all studies comparing baseline data with that during CAI. Study quality is also based on Sanson-Fisher (see methods for explanation).

‡ Schools were matched for demographics and then randomly assigned to control or intervention.

## Results

Of the 789 records originally retrieved through the indicated search terms, only fourteen matched the inclusion criteria and were included in this review^(19–32)^. Eleven out of these fourteen were conducted in the USA and one each in Denmark, Germany and Great Britain^(22,30,32)^. Four^(20,23,24,27)^ of the nine US studies were conducted as part of the smarter lunchrooms movement^(33)^.

Twelve out of fourteen studies^(19,21–23,25–32)^ compared data for students’ food choice and/or consumption collected prior to CAI implementation with that during the intervention, with one of these also reporting on student’s post-intervention food choices^(32)^ (Table 2). Out of these twelve, five reported changes in food choice within a selected school between baseline and intervention only (uncontrolled pre–post design)^(21,23,27,30,31)^. From the remaining seven, five were controlled pre–post studies^(19,22,26,28,32)^ and two cluster-randomised controlled trials^(25,29)^. Two more studies were cluster-randomised but did not report baseline data^(20,24)^. All CAI were implemented at school cafeterias or lunchrooms with one study catering for the control group at a university laboratory^(22)^. Students’ food selection was determined via sales records (Table 3)^(19,20,25,26,28,30,32)^. Food consumption was assessed either via visual observation (either directly or using photographs; quarter waste method) and/or tray/plate waste measurements^(20,23–27,29,31)^. Gross calorie and fat intake were determined by weighing selected food and beverage choices before and after service^(21)^. Frequency of school visits ranged from 1 to 17 d during the course of the CAI intervention period in eleven studies, while three used continuing sales data reports throughout the course of their respective study to calculate results^(19,28,32)^.

**Table 3:** Overview of outcomes and results of studies included in this review

Reference/study/ country	Intervention type	Measured parameters	outcomes	Main results for respective intervention period/school
Conklin MT et al. 2005^(19)^ PA, USA	Calorie and nutrient labelling of selected entrées	continuous sales data collection during each period	selection of entrées with high calories or high-fat content (> 20 g per serving)	-significant decrease in selection of high calorie entrées in comparison to control −22 % (*P *< 0·05) -non-significant difference in selection of entrées with high-fat content
Wansink B et al. 2013^(20)^ NY, USA^*^	Presentation pre-sliced apples	sales records & plate waste observation	apple sales and apple consumption	-significant increase in apple sales in comparison to control apples eaten per student +24 % (*P *= 0·10) -non-significant increase in apple consumption
Hunsberger M et.al. 2015^(21)^ OR, USA	Calorie labelling at point-of-purchase	weighing of food and beverage consumption before and after service	gross calorie intake (GCI), total fat intake	Significant decrease in gross calorie and total fat intake (pre/post) calorie intake = –41 kcal/student (*P *= 0·004) fat intake = –21 g/student (*P *= 0·0025)
dosSantos et al. 2018^(22)^ Denmark	‘Dish of the day’ labelling for the vegetable-based meal	meal choice from three different options	choice of ‘Dish of the day’	No difference in the choice of ‘Dish of the day’
Hanks AS et al. 2013^(23)^ NY, USA^*^	Labelling Presentation Position Prompting	plate waste observation	selection and consumption of fruit and vegetables	-significant increase in fruit and vegetable selection and consumption selection (pre/post) fruit: +13·4 % (*P *= 0·012); vegetable: +23 % (*P *< 0·001) consumption 1 portion (pre/post) fruit: +15·8 % (*P *= 0·006); vegetable: +9·8 % (*P *= 0·022)
Cullen KW et al. 2015^(24)^ TX, USA	Labelling Prompting	observation of selection and consumption of fruit and vegetables	selection and consumption of fruit and vegetables	-significant increase in fruit and vegetable selection in comparison to control Fruit: +24 % (*P *= 0·001) Vegetable: +11 % (*P *= 0·05) -non-significant increase in fruit and vegetable consumption
Greene KN et al. 2017^(25)^ NY,USA	Labelling Presentation Availability Position Priming	sales records and plate waste observation	selection and consumption of fruit and vegetables	-significant increase in fruit selection and consumption in comparison to control selection: +36·6 % (*P *= 0·001) consumption: +22·7 % (*P *= 0·017) -non-significant increase in vegetable selection and consumption
Quinn EL et al. 2018^(26)^ WS, USA	Labelling Presentation Position Prompting	sales records and plate waste observation	selection and consumption of fruit and vegetables	-significant increase in fruit selection in comparison to control: +9 % (*P *= 0·004) - non-significant increase in fruit consumption as well as vegetable selection and consumption
Hanks AS et al. 2012^(27)^ NY, USA	Availability Position	plate waste observation	selection of healthy food items and consumption selection of less healthy food items and consumption	-significant increase in selection of healthy food items: +18·8 % (*P *= 0·00) -no difference in consumption of healthy food items -non-significant decrease in the selection of less healthy food items -significant decrease in the consumption of less healthy food items: –27·9 % (*P *= 0·00) Diet composition changes Healthy food items share in total grams consumed: +4 % (*P *= 0·05) Less healthy food items share in total grams: –5 % (*P *= 0·00)
Boehm R et al. 2020^(28)^ Northeast, US	Labelling, Presentation, Position, Availability	food selection via sales records	sales of entrées, fruit, vegetables and milk, as well as competitive foods	-no significant increase in sales of entrées, fruit, vegetable, milk as well as competitive foods in comparison to control.
Kenney EL at al. 2015^(29)^ MA, US	Labelling, Functionality	water consumption via observation	selection and consumption of water, SSB, juice and milk at lunch period	-significant increase in selection and choice of water in comparison to control +9,4 % (*P *< 0·001) -decrease of percentage of students choosing SSB –3·3 % (*P *= 0·005) or juice –3 % (*P *= 0·03); no change in selection of milk
Winkler G et al. 2018^(30)^ Bavaria, Germany	Functionality Labelling Presentation Availability Position Priming Prompting	food selection via sales records and observations of kitchen staff	sales of fruit, vegetables, vegetarian/vegan main, whole grain, sweets, water	-fruit: significant increase (compared to baseline) at all time points phase 1: +4·1 (*P *< 0·001); phase 2(after 5 months): +7·2 (*P *< 0·001); phase 3 (after 1 year): +2·3 % (*P *= 0·069) -salad: significant increase after 5 months -vegetarian/vegan main: significant increase at last time point -whole grain: significant increase only at beginning -sweets: significant decrease observed after 5 months -water: significant increase at phase 1 and 3
Koch PA et al. 2020^(31)^ NY, US	Labelling Presentation Availability Position	selection and consumption of school lunch by photography^†^	selection and consumption of vegetables (starchy, non-starchy), fruit, grains, protein, milk as part of school lunch	-significant increase of selection and consumption of starchy vegetables (french fries) +39 % (*P *< 0·001) -decrease in consumption of non-starchy-vegetables, fruit and grains, increase in consumption of protein -no change in milk consumption
Ensaff H et al. 2015^(32)^ Yorkshire, UK	Labelling Presentation Availability Position	food selection via cashless electronic system	sales of healthy food items Vegetarian special, salads, fruits	significant increase in sales of vegetarian special, salads and fruits (pre/post at intervention school) pre = +1·4 %, intervention = +3·0 %, post = 2·2 % (*P *< 0·001 for all) 2·5 time more likely to select designated food item (*P *< 0·001)

SSB = sugar-sweetend beverages.

Light grey = studies implementing one intervention only.

Medium grey = studies reporting results for different time points during or after intervention.

* Studies from this group have provoked criticism as can be seen at https://peerj.com/preprints/3137.pdf. As a result, some studies had to be retracted. However, any study cited in this review has not been retracted at the point of writing, though results should be considered with care.

† Methodology described in Getts KM, Quinn EL, Johnson DB, Otten JJ. Validity and Interrater Reliability of the Visual Quarter-Waste Method for Assessing Food Waste in Middle School and High School Cafeteria Settings. J Acad Nutr Diet. 2017 Nov;117(11):1816–1821. doi: 10·1016/j.jand.2017·05·004. Epub 2017 Jul 6. PMID: 28688883; PMCID: PMC7261231.

None of the included studies implemented changes in sizing (Table 1), while two implemented changes in functionality^(29,30)^. The intervention type most often implemented was information/labelling (12/14), followed by changes in presentation and position/proximity (8/14 each). This preference was also observed in the four studies implementing only one intervention type^(19–22)^: three introduced specific labels (two with high calorie or fat labels, one ‘Dish of the Day’ label) and one offered pre-sliced apples, thus changing targeted product presentation. As a result of introducing calorie or fat content information, one study reported a significant decline in high fat content food sales^(19)^, while another saw a significant reduction in student’s gross calorie and fat consumption^(20)^ (Table 3). The study introducing the label ‘dish of the day’ for a vegan or vegetarian lunch alternative reported no difference in meal choice between intervention and control group^(22)^. The study offering pre-sliced apples in addition to whole ones also observed desired effects in apple sales as well as consumption, though for the latter changes were NS^(20)^.

The remaining ten studies in this review implemented two or more CAI simultaneously^(23–32)^. Four out of these compared changes in fruit and vegetable selection and consumption as part of the students’ lunch meal^(23–26)^, with all of these reporting significant increases in fruit selection and consumption during the intervention. Results for vegetable selection and consumption, though showing the desired effect by increasing, were not always significant in comparison to control or baseline. No opposing effects were reported. One study focussed on water consumption only and reported a significant increase within intervention schools after CAI^(29)^.

The remaining five studies investigated multiple CAI for a wider range of outcome parameters, including whole grain products, salads, sweets, milk and water, either recorded separately as part of the main dish, a side serving or as part of a sandwich, or included under healthy or less healthy food items or entrée^(27,28,30–32)^. Two of these recorded changes occurring during the course of the intervention by comparing students’ choice of specific food groups or consumption of school lunches at the beginning of the implementation of CAI to that of later time points^(30,31)^, while another reported on changes in food selection after interventions were removed^(32)^.

In the latter study^(32)^, changes in food environment were implemented for 6 weeks (intervention period) during the summer term 2013/14 before the baseline state of the school canteen was reinstated (post period), i.e. all interventions were removed. During the intervention period, sales of designated food items (vegetarian specials, salads and fruits) increased significantly by 3 % and stayed up by 2·2 % in comparison to baseline for the 3 weeks post period even after the removal of all CA changes. In contrast, CAI remained in place in the other two longer-term studies^(30,31)^. In the German study, sales of designated food items were observed in comparison to baseline (phase 1), right after the introduction of changes (phase 2), after 5 months (phase 3) and after 1 year (phase 4) on selective days. Sales in fruit rose by 4·1% to 7·2 % before decreasing to 2·3 % in the last phase of the study – still significantly higher than baseline sales. Though results for all other food items investigated showed significant changes in the intended direction at some phase of the study, results were less conclusive overall. For some food opposing effects to those intended were reported: e.g. sales in vegetables and sweets decreased during phase 2 as did sales for the vegetarian/vegan main dish during phases 2 and 3 before increasing again in a later phase (Table 3)^(30)^. A similar design was employed by the US-based study where consumption of different food groups as part of a school lunch menu was observed pre-, 3 months and 1 year after a redesign of the schools’ cafeteria. While non-starchy vegetable consumption increased slightly at the 3-month point, an overall decrease was reported after 1 year. This was also seen for grains and fruits consumption, while white potato and protein consumption increased overall.

## Discussion

This systematic review focussed on the target group of healthy adolescents in a secondary school setting while evaluating the impact of implemented CAI depending on type and number of implemented interventions as well as possible sustained effects.

We identified fourteen papers reporting results of one or more CAI according to the TIPPME typology^(8,9)^, indicating the promotion of healthier eating for our relevant target group in a secondary school setting. The majority of studies included in this review (10/14) implemented more than one CAI at the same time (Table 3). However, multiple changes in food environment do not seem to be essential for success, as three of the four studies implementing only one CAI reported significant changes in food choices for their respective targeted food items in the intended direction^(19–21)^. Due to the limited number of papers reporting results for the implementation of a single change in the cafeterias’ food environment as well as intervention types implemented, no conclusions can be drawn so far with regards to the effectiveness of one intervention type compared to another, or how several interventions might interact for this setting. Both intervention types implemented alone – labelling or presentation – produced significant changes for at least some of the measured parameters without causing any opposite effects. However, while concurrent calorie as well as nutrient content labelling at point-of-purchase did decrease the selection of high-fat entrées in one study, the selection of high-calorie entrées was not affected by that change^(19)^. In contrast, by adding only a calorie label to different lunch menus in a similar approach, researchers reported a decrease in gross calorie intake and an associated significant reduction in total fat intake^(21)^. These results suggest that fewer, but well-aimed, labelling approaches might actually prove more effective than more extensive ones, and that any change in the students’ food environment should be clearly targeted and based on desired aims.

In addition, the impact of one or multiple CAI might be enhanced by assessing the reasons for not choosing certain healthier food items prior to implementing any changes in the food microenvironment. One study reported that one reason why whole apples were only chosen rarely by students as part of their lunch menu was the difficulty in eating them, especially for students with braces^(20)^. By providing pre-sliced apples, a significant increase in apple sales was observed though consumption did not increase to the same extent. Another study focussing on increasing students’ water consumption from existing water fountains in the lunch room achieved its goal by providing information about the safety and benefit of the provided tap water in addition to offering free, reusable drinking cups as part of its CAI, thus specifically addressing concerns and reservations within the student population^(29)^. However, any kind of prior questioning of the student population towards their food preferences could bias them, as adolescents do have the tendency to comply with what they think is expected of them^(34)^ thus leading to a form of social desirability bias. Previous studies have shown that students are in general aware about what healthier food choices are and what they are expected to choose^(35)^.

Taken together, these results suggest that any CAI should be implemented with special emphasis towards the aims regarding the selected target food group, as even single well-chosen and placed changes can promote effective positive changes in food choices. Future research should be designed to investigate the impact of other intervention types when implemented alone to determine parameters that foster a successful implementation for each type independently. Students’ opinions and attitudes should also be further investigated.

Another area of future interest should be the determination of lasting effects of any CAI implemented, as some of the immediate changes in food choices could primarily be the result of some form of novelty effect^(11)^. Only three out of the fourteen papers meeting the inclusion criteria for this review were designed to investigate the sustained impact of the changes implemented on the food environment within the investigated student population^(30–32)^. The major difference between these three studies was the removal of all intervention measures in the UK-based study at the end of the intervention period^(32)^, whereas interventions remained in place in the other two studies conducted in Germany and the USA, respectively^(30,31)^. Results from these two studies indicate that permanent changes in the food microenvironment do not necessarily guarantee continuous positive effects. Though changes overall were encouraging in the study conducted in Germany^(30)^, they were less clear cut, with some of the food groups investigated showing opposing effects in sales in comparison to control at certain time points over the course of the intervention year. Similar results were also reported for the US-based study^(31)^ where consumption of non-starchy vegetables as well as fruits and grains as part of the students’ school lunch decreased after 1 year of CAI, despite showing promising results after 3 months. Possible explanations offered by the authors were, amongst others, seasonal changes in food preferences by the students, changes and general challenges within the organisational set-up as well as presentation and promotion of certain foods offered in the course of the school year, combined with some habituation effect towards the no longer ‘new’ changes.

These results illustrate the need for further (long-term) investigations into the potential factors influencing food choice and eating habits in a school environment, despite the practical challenges of such investigations in a working environment. Some information in that respect was generated as part of the US-based study as students’ attitudes towards certain factors in the cafeterias were also investigated by attitude scales. While attitudes were highest at the 3-month post-redesign time point, most of them eventually showed no change from baseline after one year. These findings underline that food environments are not static and that the needs of certain target populations are constantly changing with respect to that environment, making it necessary to adjust interventions accordingly to achieve continuous effects. Such adjustments might even include the removal of all intervention measures after a CAI as reported by the UK-based study. Overall positive results in increasing favourable food choices continued, even after the removal of the intervention measures after 6 weeks. Though sales for favourable food items did decline after the removal of CAI in comparison to sales during the intervention period, they were still significantly higher than at baseline.

Taken together, more long-term studies preferably with higher evidence-level designs are needed, which also investigate other external factors affecting adolescent’s food choice. Comparable conclusions are also drawn by Metcalf et al^(36)^ calling for more research with regard to the impact of individual interventions as well as other factors influencing food choice during school meals. This group investigated nudging across all school forms and also highlighted the difference between food choice and actual food consumption by students. Though a positive association between CAI and food choice is described in their synthesis, conclusive data and methodology for the investigation of consumption of the chosen foods are often lacking. This should also be considered for future long-term studies, especially with regard to the sustainability not only of CAI but also regarding the overall sustainability of meal offers at schools.

Despite the promising findings, one also has to consider the limitations of this review. Only two (though large) databases were searched for this review, yielding the fourteen studies included in this synthesis. Of these, only four can be rated as having a strong quality according to Sanson-Fisher^(37)^, i.e. they were cluster randomized controlled trials, while the remaining ten would be rated as moderate or moderate-to-weak due to controlled or uncontrolled pre–post study design (Table 2). Moreover, only a limited number of studies matched the inclusion criteria, to begin with, and of those, heterogeneity in study design, outcomes, reporting and target foods as well as CAI implemented further restricted our ability to quantify and assess the overall effects of the different interventions. On the other hand, this review is the first to focus on the target group of students in a secondary school setting rather than certain specific food groups, thus providing a more general overview of how to successfully promote changes in this particular age group.

## Conclusion

This review found encouraging evidence for the effectiveness of CAI on food selection and consumption of healthy adolescents in a secondary school setting, with all except one study reporting overall positive changes in the desired direction. Neither the number nor types of CAI implemented seemed to be a decisive factor for success. In contrast, long-term studies suggest the needs and demands of the target group should be considered more closely, and CAI should be adjusted over time for the respective food items they are promoting. However, for more differentiated conclusions, more cluster-randomized controlled trials, i.e. designs with a higher evidence level, are needed aimed at investigating not only the influence of CAI types and numbers but also other external factors on adolescent’s food choice over the course of at least one school year.
